# Diabetic retinopathy screenings in West Virginia: an assessment of teleophthalmology implementation

**DOI:** 10.1186/s12886-023-02833-4

**Published:** 2023-03-10

**Authors:** Travis Schofield, Ami Patel, Joel Palko, Ghassan Ghorayeb, L. Carol Laxson

**Affiliations:** Department of Ophthalmology and Visual Sciences, West Virginia University School of Medicine, Morgantown, WV 26506 USA

**Keywords:** Diabetic retinopathy, Teleophthalmology, Diabetes, Telemedicine, Retinal screening, Fundus photography

## Abstract

**Background:**

The prevalence of diabetes in the state of West Virginia (WV) is amongst the highest in the United States, making diabetic retinopathy (DR) and diabetic macular edema (DME) a major epidemiological concern within the state. Several challenges exist regarding access to eye care specialists for DR screening in this rural population. A statewide teleophthalmology program has been implemented. We analyzed real-world data acquired via these systems to explore the concordance between image findings and subsequent comprehensive eye exams and explore the impact of age on image gradeability and patient distance from the West Virginia University (WVU) Eye Institute on follow-up.

**Methods:**

Nonmydriatic fundus images of diabetic eyes acquired at primary care clinics throughout WV were reviewed by retina specialists at the WVU Eye Institute. Analysis included the concordance between image interpretations and dilated examination findings, hemoglobin A1c (HbA1c) levels and DR presence, image gradeability and patient age, and distance from the WVU Eye Institute and follow-up compliance.

**Results:**

From the 5,512 fundus images attempted, we found that 4,267 (77.41%) were deemed gradable.  Out of the 289 patients whose image results suggested DR, 152 patients (52.6%) followed up with comprehensive eye exams—finding 101 of these patients to truly have DR/DME and allowing us to determine a positive predictive value of 66.4%. Patients within the HbA1c range of 9.1-14.0% demonstrated significantly greater prevalence of DR/DME (*p* < 0.01).  We also found a statistically significant decrease in image gradeability with increased age.  When considering distance from the WVU Eye Institute, it was found that patients who resided within 25 miles demonstrated significantly greater compliance to follow-up (60% versus 43%, *p* < 0.01).

**Conclusions:**

The statewide implementation of a telemedicine program intended to tackle the growing burden of DR in WV appears to successfully bring concerning patient cases to the forefront of provider attention.  Teleophthalmology addresses the unique rural challenges of WV, but there is suboptimal compliance to essential follow-up with comprehensive eye exams. Obstacles remain to be addressed if these systems are to effectively improve outcomes in DR/DME patients and diabetic patients at risk of developing these sight-threatening pathologies.

## Background

Among working age adults, diabetic retinopathy (DR) is the most frequent cause of blindness [1]. Progression to eye pathology can be rapid, with nearly 100% of type I diabetes patients and > 60% of type II diabetes patients presenting with DR within the first two decades of diagnosis [2]. It has been nationally estimated that 28.5% and 4.4% of diabetic patients in the U.S. have DR and vision-threatening retinopathy, respectively [3]. While this is certainly a national concern with about 34.1 million American adults being diagnosed with diabetes, West Virginia (WV) has the highest prevalence of diabetes (16.2% as of 2018) [4, 5]. The state also faces unique challenges given its predominantly rural setting (over 37% of its population designated as rural in comparison to 14% of the total U.S. population) [6, 7]. The rural challenges of WV are compounded by the state’s notably high rates of poverty, unemployment, and low education [8, 9]. In hopes of circumventing some of these challenges, clinicians have turned to novel approaches like telemedicine in order to provide WV’s diabetic population with improved care. Teleophthalmology is one such approach and serves as the foundation for the investigations of this study.

Primary care offices may be more accessible to patients than those of specialists, especially in rural locations. Trained nurses and staff at these locations use cameras to acquire fundus photographs that can be uploaded for review by off-site specialists. Although there are limitations to the single-field, nonmydriatic fundus photography implemented at these primary care sites, these tools have allowed for detection of eye pathology in a variety of settings [10, 11], and it has been proven to be a sensitive screening tool for retina pathology, such as DR and diabetic macular edema (DME) [12]. Hence, teleophthalmology systems have been emplaced within the West Virginia University (WVU) Hospitals system. Using the U.S.-Food-and-Drug-Administration-approved Intelligent Retinal Imaging Systems (IRIS), primary care offices throughout the state have incorporated teleophthalmology into their clinical practice. Utilizing data acquired via teleophthalmology, ophthalmologists of the WVU Eye Institute have been enabled to provide guidance across the state based on their assessments of images acquired at these remote locations.

The aim of this study is to assess the success and shortcomings of WV’s teleophthalmology implementation by analyzing data regarding image gradeability and concordance between photographic screenings and subsequent comprehensive eye exams in clinic. While studies have shown that teleophthalmology is effective in assessing retina pathology and guiding appropriate referral decisions [12], we utilize this opportunity to assess the use of this technology specifically within WVU Medicine and its affiliates. Different screening modalities have been explored in the literature (nonmydriatic versus mydriatic screening [13, 14], varying fields of view [15], artificial intelligence systems [11], and smartphone-based retinal photography [13, 16]). Given that nonmydriatic, 45-degree photography was utilized in screening our population, we were interested in comparing our findings to that which has been observed and reported through other telehealth programs [17, 18]. With 20.9% of WV’s population being over 65 years, we were also interested if age would play a role in the gradeability of images obtained during screening [19].

While diabetic retinopathy can be vision-threatening, proper management of diabetes and ophthalmic interventions like pan-retinal photocoagulation (PRP) and intravitreal anti-vascular endothelial growth factor (anti-VEGF) agents have shown to be effective and have become the current standard of care in managing diabetic retinopathy at various stages of its progression [20]. Hemoglobin A1c (HbA1c) severity and the presence of hyperreflective spots on spectral domain optical coherence tomography (an indicator of diabetic retinopathy progression) has shown to be linear with any HbA1c over 5.4% demonstrating a high likelihood of presenting with hyperreflective spots [21]. Therefore, we have utilized the opportunity of this retrospective chart review to investigate this correlation and to determine how it might be reflected in the process and outcomes of this screening modality.

Expansion and improved accuracy in screening modalities holds substantial promise as the burden of diabetes continues to increase across the country. However, the success of these screening programs in facilitating appropriate care for patients under suspicion for vision-threatening diabetic retinopathy heavily relies on patient compliance to their providers’ recommendations. Numerous factors can affect patient compliance to care plans for diabetic retinopathy, including age, education, duration of their diabetes, practical understanding of their condition, and understanding/communication of the purpose behind teleretinal screenings [22, 23]. Given the rural setting of WV, we also sought to explore how the geographic boundaries might impact patient follow-up, which is essential to the ultimate success of these screening programs [22].

## Methods

This retrospective medical chart review consisted of collecting data regarding diabetic patients 18 years and older who have participated in the teleophthalmology program offered throughout the state of WV between January 2017 and June 2019. The WVU institutional review board approved the study protocol. The Volk Pictor (Volk Optical, Inc., Mentor, OH, USA) nonmydriatic cameras used by trained nurses and staff acquired 45-degree fundus images from patients at various primary care and endocrinology clinic settings. In these settings, patients waited in rooms with the lights turned off to maximize pupillary dilation sans mydriatic drop administration. Staff would use the handheld fundus cameras to take photographs that were then uploaded and subsequently reviewed by retina specialists. Both eyes were photographed when possible with hopes of acquiring at least one viable image per eye. The number of attempts made was contingent on the judgment of the trained staff acquiring the images and the tolerance demonstrated by the patients being screened for repeated attempts.

**Fig. 1:**
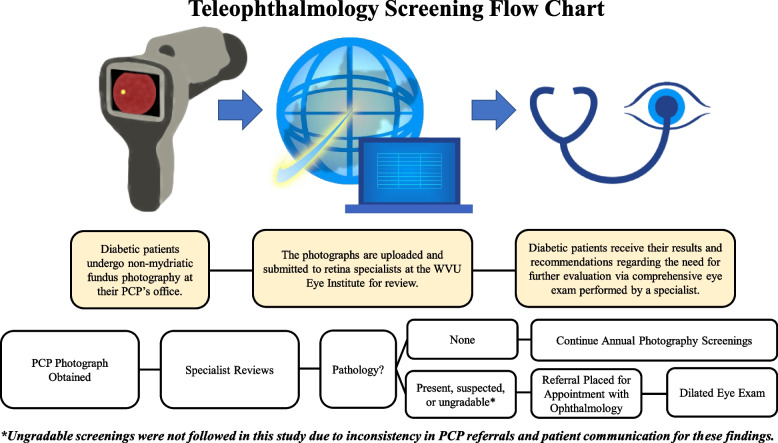
Teleophthalmology flow chart

Images were graded by a retina specialist at the WVU Eye Institute. These specialists included three WVU board-certified retina faculty and one vitreoretinal fellow—all patients were assigned to have their set of acquired images evaluated by one of these four specialists. Images were noted as gradable or ungradable, and the extent of DR (absent, mild, moderate, severe, or proliferative) and/or DME (absent, mild, moderate, or severe) was described in accordance to the International Classification of DR scale [24]. Care plan recommendations and suspicion of other pathologies were also noted. The results with their accompanying care plan recommendations were uploaded to the Epic electronic medical record (EMR) for the use of primary care physicians (PCPs) in their advising of diabetic patients in accordance to the American Academy of Ophthalmology’s guidelines for DR follow-up (Fig. 1). Referral recommendations were made in accordance to those proposed by the International Council of Ophthalmology (ICO) and American Diabetes Association (ADA) [25]—albeit with the decision to recommend referral for suspected DR of any severity. Recommendations could also be made on the basis of other ocular pathologies that were remarked by reviewing ophthalmologists (e.g., age-related macular degeneration, choroidal nevi, colobomas, hypertensive retinopathy, glaucomatous optic nerves). For the purpose of this study, we exclusively followed patients whose screening findings indicated suspicion for diabetic retinopathy of any severity in at least one eye.

### Data collection

Lists of photography instances were generated, and these lists were used to investigate all photography orders recorded in the Epic EMR utilized by WVU Hospitals between January 2017 and June 2019. Photography orders that were unfulfilled (due to premature order placement by clinicians, for instance) were excluded from the study. Patient information was de-identified, and spreadsheets in Microsoft Excel were created to collect and organize the data.

Each valid photography order was investigated in the following fashion. First, the IRIS results adjoined to patients’ charts for the photography order in question would be accessed. The gradeability and presence of pathology would be recorded (specifically noting DR as mild, moderate, severe, or proliferative and DME as mild, moderate, or severe). If the screening results indicated suspicion for pathology, further investigation was conducted. Date of birth, the time that had passed since their diabetes diagnosis, their diabetes classification (Type 1 or Type 2), and HbA1c within 3-months of their photography date were all collected. Patient receipt of their results (either through record of PCP communications or indications that patients had read their results via the patient-accessible WVU MyChart system) was recorded, and whether or not an appointment was subsequently set and maintained (within 12 months of the photography order date or prior to a future repeat screening with their PCP) was also noted. Using patients’ home addresses, distances from the WVU Eye Institute to patients’ hometowns were recorded using Google Maps driving estimates. The results of patients’ dilated eye exams were recorded (noting severity as mild, moderate, severe, or proliferative for DR and absent or present for DME). Where feasible, these data were acquired from offices outside of WVU Medicine by either viewing documentation that had already been uploaded to the Epic EMR by patients or their providers or by contacting these offices directly where references in PCP notes indicated completion of ophthalmic follow-up outside WVU Medicine and permission had been granted.

### Statistical analysis

Using the data review functions of Microsoft Excel, summations and calculations were performed with the data acquired from the 2,756 patients who were studied using teleophthalmology within our selected timeframe. The totals and percentages of each attribute of interest were calculated—gradeability and the totals and proportions of DR/DME severities in PCP screenings and subsequent dilated eye exams. Pearson’s chi-squared tests were performed to compare the gradeability data found within different age ranges (18–49 years, 50–64 years, and ≥ 65 years) and the prevalence of DR within different HbA1c ranges (5.4–6.4%, 6.5–9.0%, and 9.1–14.0%). This method was also used to investigate the relationship between patient distance from the WVU Eye Institute and compliance to follow-up with dilated eye exams.

## Results

**Fig. 2:**
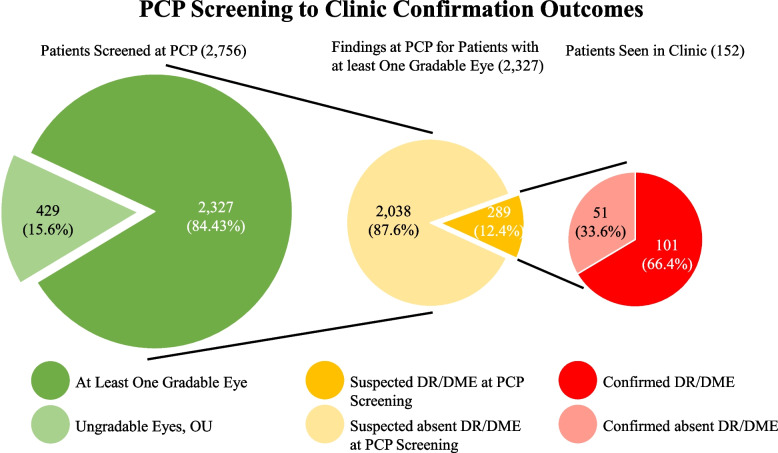
PCP Screening to clinic pipeline with patient outcomes

Through WVU Medicine’s teleophthalmology screenings, 2,756 patients received screenings between January 2017 and June 2019 (first order date: 01/12/2017, last order date: 06/28/2019). From these 2,756 patients, 2,327 photography results (84.43%) were deemed to possess at least one gradable eye by retina specialists at the WVU Eye Institute. Both eyes were deemed gradable in 1,940 patients (70.39%). Two hundred, eighty-nine patients (12.4% of the patients with at least one gradable eye) had results noting some form of DR or DME. These patient cases were explored further, and it was found that 152 of these patients followed up in clinic within 12 months of their screening or prior to receiving another nonmydriatic screening with their PCP (124 within WVU Medicine, 28 with outside/external ophthalmologists). DR/DME was confirmed in 101 of these patients (Fig. 2). The confirmation of true positives with dilated eye exams enabled a calculation of the screening method’s positive predictive value. The positive predictive value was calculated to be 66.4% for the teleophthalmology screening’s capacity to detect true DR/DME pathology. Other pathology notes and specifiers were also recorded, and 114 instances of age-related macular degeneration, 43 instances of hypertensive retinopathy, 60 instances of glaucomatous optic nerves, 17 instances of choroidal nevi, 3 instances of dot and blot hemorrhages, and 1 case of chorioretinal scar versus coloboma were noted throughout the data collection of screening results and subsequent dilated eye exam findings.

**Fig. 3:**
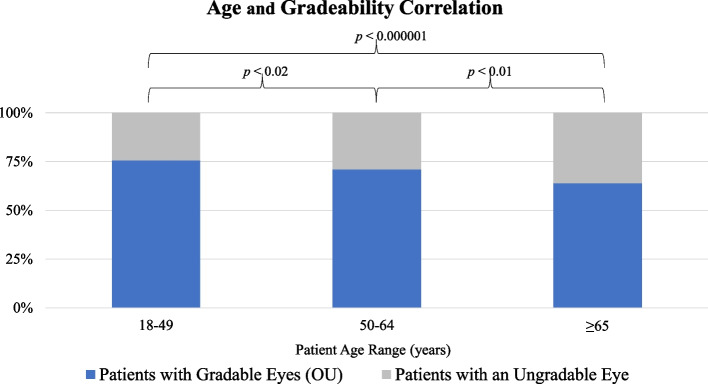
Increasing ungradable fundus images with age

The gradeability of the screening photographs varied by age in that patients aged 65 years and older were found to have statistically significantly fewer gradable eyes than patients younger than 65 years (63.9% versus 72.7%, respectively, *p* < 0.00001), and the mean age was found to be 57.97 years (*σ* = 12.66) (Table 1). Breaking the < 65 years of age group down further reveals a statistically significant difference between the age ranges of 18–49 and 50–64 (75.6% versus 70.9%, respectively, *p* < 0.02), 18–49 and ≥ 65 (75.6% versus 63.9%, respectively, *p* < 0.000001), and 50–64 and ≥ 65 (70.9% versus 63.9%, respectively, *p* < 0.01) (Fig. 3).

**Table 1:** Gradeability and age analysis

Date Range	**01/2017–06/2019**	
Total number of patients with photography	2,756	
Total number of patients with at least one gradable eye	2,327 (84.43%)	
Mean Age	57.97 years (*σ* = 12.66)	
Age Ranges	Total Patients	Gradable Eyes (OU)
18–49 years	783	592 (75.6%)
50–64 years	1,256	890 (70.9%)
≥ 65 years	717	458 (63.9%)

**Fig. 4:**
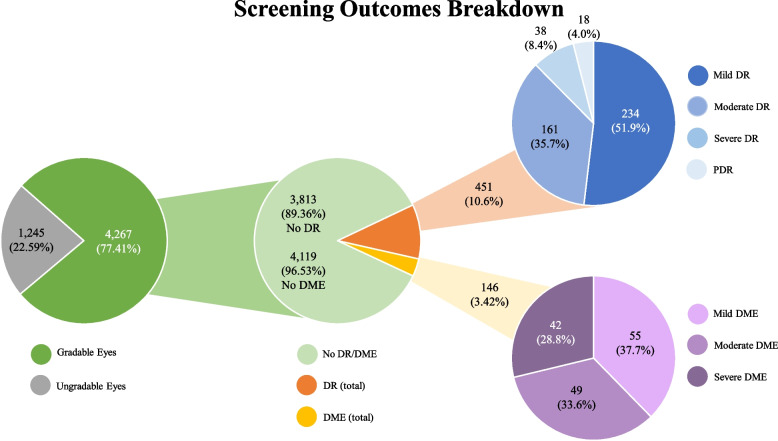
Eye Findings as per PCP screening

With 2,756 patients screened, 5,512 eyes were attempted to be screened. However, 4,267 eyes (77.41%) were deemed gradable, and 1,245 eyes (22.59%) were deemed ungradable. No suspicion for DR was raised in 3,813 eyes (89.36%), and 4,119 eyes (96.53%) did not raise suspicion for DME. Some severity of DR was described in 451 eyes (10.6%), and 146 eyes (3.42%) showed some severity of DME. The majority of DR cases, 234 eyes (51.9%), were described as mild. Moderate DR was described in 161 eyes (35.7%), and 38 eyes (8.4%) were described to demonstrate severe DR. PDR was noted in 18 eyes (4.0%). Mild DME was described for 55 eyes (37.7%), moderate DME was described for 49 eyes (33.6%), and severe DME was described for 42 eyes (28.8%) (Fig. 4).

Patient cases with DR/DME pathology were further investigated. Some note of an appointment being set was indicated in the EMR for 170 patients. One hundred, nine patients had record of an appointment being set within three months of their screening, 12 patients had record of an appointment being set within six months of their screening, 17 patients had record of an appointment being set within 12 months of their screening, and 16 patients were noted to have had an appointment set beyond 12 months but prior to their next screening with their PCPs. It was found that some form of PCP follow-up occurred in 272 cases, or 94.1% of the patient cases in which DR/DME pathology was noted. PCP follow-up was deemed to have occurred if there existed some recorded form of communication between the PCP and the patient in the EMR (e-mail, phone conversation, WVU MyChart messages, et cetera) or if it was indicated that the patient had viewed their results on WVU MyChart. Compliance with follow-up varied depending on patient hometown distance from the WVU Eye Institute. It was found that patients who resided within 25 miles demonstrated statistically significantly greater compliance to follow-up with a dilated eye exam than those residing farther than 25 miles away (60% versus 43%, respectively, *p* < 0.01) (Table 2). As mentioned previously, 28 of the 152 patients who followed up in clinic were found to have records available regarding their follow-up appointments for a dilated eye exam with an ophthalmologist outside WVU Medicine. Outside appointments made up 3% of the follow-up visits for those residing within 25 miles and 16% of the follow-up visits for those residing beyond 25 miles.

**Table 2:**
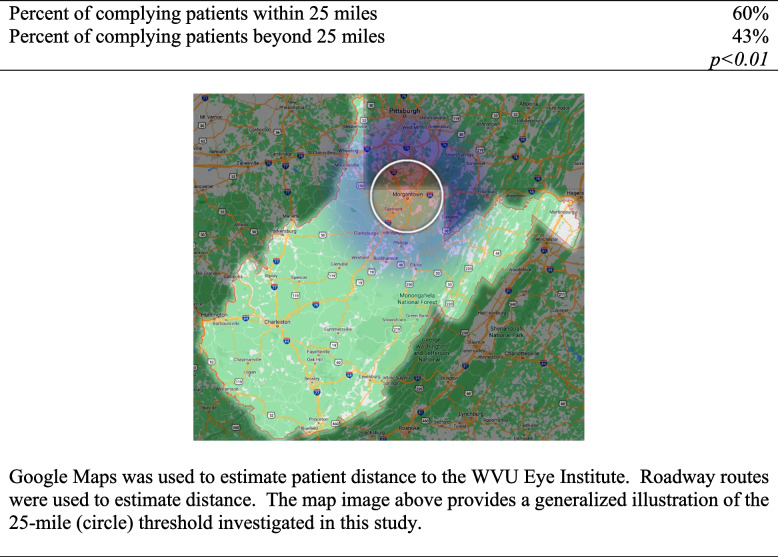
Patient distance and follow-up compliance

This patient data includes all patients with DR/DME noted in photography results (including those who complied to follow-up at external/outside offices where information was available)

**Fig. 5:**
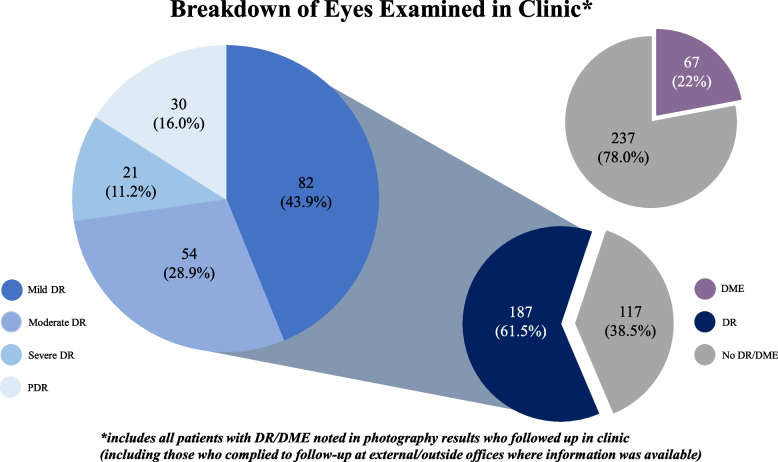
Eye findings as per comprehensive eye exams in clinic

Data collected from the follow-up exams revealed 187 eyes (61.5%) with DR and 67 eyes (22%) with DME. The majority of eyes with confirmed DR had mild DR (82 eyes, or 43.9%). Fifty-four eyes (28.9%) were diagnosed with moderate DR, 21 eyes (11.2%) were diagnosed with severe DR, and 30 eyes (16.0%) were diagnosed with PDR. The presence of DME was found in 67 eyes (22%) (Fig. 5).

**Fig. 6:**
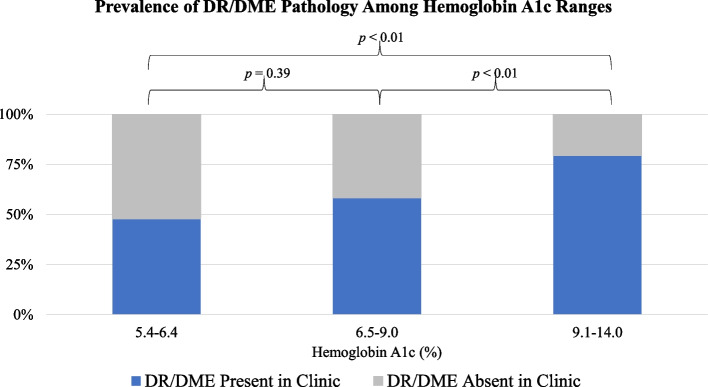
DR/DME Prevalence Among HbA1c Ranges

Regarding patient diabetes status of those under suspicion for DR/DME pathology in their initial screenings, 91% were diagnosed with type 2 diabetes, and 9% were diagnosed with type 1 diabetes. The mean duration of diabetes (determined by calculating the time transpired between the date of the patient’s photography and the earliest mention of a diabetes diagnosis or a historical account of such a diagnosis predating the EMR) was 6.8 years (*σ* = 5.3). The mean HbA1c was calculated to be 8.9% (*σ* = 2.2) (Table 3). When the prevalence of DR/DME pathology (confirmed in clinic via dilated eye exam) was compared among patients falling within three ranges of HbA1c levels via Pearson’s chi-squared tests, no statistically significant difference was found when comparing the 5.4–6.4% range to the 6.5–9.0% range (*p* = 0.39). However, a statistically significant difference was appreciated in comparison between the 5.4–6.4% range and the 9.1–14.0% range (*p* < 0.01) and between the 6.5–9.0% range and the 9.1–14.0% range (*p* < 0.01) (Fig. 6).

**Table 3:** Patient results and concordance of findings

Total number of patients with DR/DME via teleophthalmology	**289**
Mean HbA1C	8.9%
	*σ* = 2.2
Type I diabetes prevalence	9%
Type II diabetes prevalence	90%
Mean duration of diabetes^a^	6.8 years
	*σ* = 5.3 years
Total number of patients with DR/DME (according to comprehensive eye exam)	101 (34.9%)
Positive Predictive Value	66.4%

^a^Time between documented diagnosis and photography date

## Discussion

The success of an implemented teleophthalmology screening program is contingent on both the screening process and subsequent patient compliance to recommendations based on the screening results. Through this retrospective chart review concerning WV’s teleophthalmology program, we have come to identify several areas of interest for improving our understanding of the screening process and its outcomes in this population.

The first essential step of these programs is the acquisition of the fundus images. Accurate assessment is reliant on the successful attainment of gradable fundus photographs. Our investigation revealed that 84.43% of the 2,756 patients screened had at least one gradable eye, but both eyes were gradable in only 70.39% of cases. The proportion of images acquired and deemed gradable in our cohort was comparable to previous studies. For instance, Tarabishy et al. found that 95.1% of the 1,175 patients from which they acquired 45-degree images with a nonmydriatic camera to have gradable eyes [26]. Benjamin et al., however, acquired 1,377 45-degree images via nonmydriatic fundus photography and found that 67.4% were gradable [27]. Several factors may play a role in this variability of gradeability outcomes among studies. How photographers are trained, what equipment is utilized, and whether or not dilation is an option are all factors that need to be considered. Additionally, individual variation in photographers’ thresholds for the number of attempts they make in acquiring images and the number of attempts a patient may be willing to tolerate during their PCP visits may also influence these results. Time and resources at PCP offices are also likely to vary. While the equipment, guidelines, and absence of dilation were consistent among our screenings, these other factors are more elusive and may very well have impacted the outcomes we observed. Additional standardization and documentation of the imaging protocol would provide insight into the limitations of the current screening methodology.

There is also the question of image grader variability. The concern of variability for deeming images gradable or ungradable could also be extrapolated to the image interpretations and the use of modifiers describing the extent of DR/DME observed. A limitation of this study was the use of single graders to evaluate the images—precluding the calculation of an inter-observer correlation. While we could not explore this aspect further in our own work, previous telemedicine investigations like those conducted by Liu et al. in 2019 have provided reassurance—concluding that, while it is recommended that uniform standards be established to improve consensus on image gradeability, it is unlikely that there exists much variability among ophthalmologists when assessing diabetic retinopathy through these screening methods [28].

When patients’ screening results were found to raise suspicion for DR/DME, their cases were explored further to analyze concordance with subsequent dilated eye exam findings. However, in order to confirm the diagnosis, patients first needed to comply with the recommendation to see a specialist. We found that out of the 289 patients who had DR/DME pathology noted on their screening results, only 152 (52.6%) complied with subsequent appointments for a dilated eye exam. This seems to be a problem appreciated among other teleophthalmology programs as well. The investigations reported by Bresnick et al. in 2020 noted the effectiveness of these screening modalities in identifying patients in need of further examination and possibly specialized care. However, they recognized that about half of their patients failed to keep their first ophthalmology appointments and have, hence, initiated the implementation of a tracking/recall system to ensure that these at-risk patients do not miss this potentially crucial step in their vision care [29].

We attempted to explore this aspect by investigating follow-up by PCPs. While we found that 94.1% of patients with noted DR/DME pathology on screening received some form of notice regarding their results, the notification method and content varied widely. Some patients received phone calls, e-mails, or WVU MyChart messages from their providers with explanations of their results and the appropriate next steps in their care. Others merely looked over the results themselves once uploaded and made viewable on WVU MyChart—possibly without any further explanation of what the results mean for their care. Some patients set appointments but failed to adhere. Some never made appointments, and other charts contained notes suggesting that an outside ophthalmologist or optometrist was planned to be seen with regards to their vision care in general. The variability in this crucial step of teleophthalmology may have contributed to the lack of compliance we observed. Furthermore, we observed that follow-up recommendations varied by PCP. While the ICO/ADA guidelines suggest referral for any cases in which photographs cannot be adequately obtained or assessed [25], we noticed a discrepancy in how PCPs managed these results. As mentioned previously, some communication of the results was sparse—some patients only seeing that their results were deemed ungradable in WVU MyChart. Other PCPs directly contacted their patients in some way, but sometimes repeat screening was chosen over referring patients to specialist care. While we chose to focus this study on the follow-up of positive screenings, this is indubitably concerning and warrants intervention for improved adherence to protocol and limiting the number of potential DR suspects who may be missing opportunities for diagnosis confirmation and subsequent care. When patients with suspected DR/DME did comply with follow-up, we found that 101 patients truly had DR/DME of various severities. While our study was limited in that we lacked the true and false negative data to explore sensitivity and specificity like previous studies [26] (we did not have patients who screened negative report to clinic for dilated eye exams for confirmation), we were able to determine that the positive predictive value of our screening was 66.4%.

One variable we anticipated having an impact on the gradeability results was patient age. As mentioned previously, a notably high proportion of WV’s population is aged 65 years and older [19]. The state of WV also demonstrates the greatest prevalence of diabetes [5]. With these details in mind, it was suspected that age could influence the outcomes of this study. Through our investigations, we found that there was a statistically significant difference in image gradeability between patients aged 65 years and older and those aged younger than 65 years (63.9% versus 72.7%, respectively, *p* < 0.00001). We suspect that this may be related to other ophthalmic changes commonly associated with the aging eye. For instance, refractive status and cataract development could impact the clarity of the images obtained. Nonmydriatic cameras were utilized in our screenings—attempts to maximize pupillary dilation only being achieved by having patients wait in a dark room prior to screening. Given that pupillary diameter is known to decrease with age, this could have contributed to the significant difference in gradeability we observed among patient screenings in this population [30]. Further research is required to determine if dilation in more elderly populations would substantially lower the rate of ungradable images. Interestingly, stratifying this data further into three age ranges further elucidates a negative correlation between age and image gradeability. We found a statistically significant difference between the age ranges of 18–49 and 50–64 (75.6% versus 70.9%, respectively, *p* < 0.02), 18–49 and ≥ 65 (75.6% versus 63.9%, respectively, *p* < 0.000001), and 50–64 and ≥ 65 (70.9% versus 63.9%, respectively, *p* < 0.01).

Other relevant details were explored for patients with pathology noted on screening in order to compare to previously observed trends. For instance, HbA1c severity has been shown to correlate with indicators of diabetic retinopathy severity and has served as a useful biomarker of chronic hyperglycemia, and blood glucose control has been shown to improve outcomes for retinopathy [20, 21, 31]. With this in mind, we divided patients with suspected DR/DME pathology into three HbA1c categories: 5.4–6.4% to represent the prediabetes range (diabetic patients with presumably better glycemic control), 6.5–9.0% to represent a mid-range, and 9.1–14.0% to represent the most severe cases. While we did not find a statistically significant difference between the 5.4–6.4% and 6.5–9.0% ranges (*p* = 0.39), both of these ranges demonstrated a statistically significant difference when compared to the 9.1–14.0% range (*p* < 0.01, *p* < 0.01). Our mean HbA1c was 8.9% (*σ* = 2.2). These findings together seem to align with previous studies [27].

The false negative data was unfortunately not available for our study. Since we retrospectively studied a real-world application of teleophthalmology in which it was not recommended for patients to pursue ophthalmic follow-up for negative screenings [25], we were unable to confirm the true and false negatives. Previous studies, however, reveal data not dissimilar to those found in our study—demonstrating a greater proportion of absent or mild DR than more severe cases [32]. Nevertheless, this does not make it possible to extrapolate true and false negative rates. Furthermore, our prevalence of DR/DME by screening is notably lower than expected when compared to pooled prevalence data reported in the literature. Globally, the prevalence of DR has been estimated to be 22.27% [33]—some studies estimating as high as 34.6% [34]. Our screening raised suspicion for DR/DME in only 12.4% of patients (with at least one gradable eye), and capturing an accurate prevalence of DR in our population is challenged further as only a subset of these patients maintained follow-up to confirm their diagnoses. However, variation in this prevalence data appears to be commonly reported among individual studies and population subgroups [33, 34]. These variations may be explained by aspects as technical as the differences in screening modalities or as fundamental as the patient demographics. Variables expected to influence the prevalence data include major risk factors, such as duration of diabetes and HbA1c [34]. Our mean HbA1c and mean age, however, suggest these factors are less likely to be contributing to the lower-than-expected DR prevalence we observed since they bear semblance to those of other studies [27]. According to the findings reported by Sato et al., our mean duration of diabetes also suggests there was ample time for expected progression to PDR [35].

It has also been reported that there is a significant difference in DR prevalence among different races—with a significantly higher DR prevalence in blacks and Hispanics (36.7% and 37.4%, respectively) compared to whites (24.8%) [36]. There also appears to be intra-ethnic variation. For instance, Yau et al. reported a significantly higher prevalence of DR in a U.S. Caucasian population compared to an Australian Caucasian population (35% versus 15.3%) [34]. Genetic and environmental risk factors may all play a role in disease progression and management, and these variations may render it difficult to assess whether the DR population of WV is sufficiently being addressed. However, they may also suggest that some variation is to be expected with the unique genetic and environmental makeup of a population. Unlike past studies with greater representation of ethnic minorities [18, 27, 28], 93.1% of WV’s population is white [8]. Additionally, the state’s rural setting and notably high rates of poverty, unemployment, and low education could impact the screening and subsequent follow-up on which this prevalence data relies [8, 9]. Interestingly, a study reported an unexpected lack of association between low socioeconomic status and higher grades of DR, which could be relevant to the socioeconomic impact in our WV population [37]. Ultimately, it is difficult to pinpoint whether our lower-than-expected prevalence is due to false negative screenings, ungradable images of patients with DR, or selection bias of our retrospective cohort.

While we are unable to explore the true and false negatives and this is undoubtedly a valuable component in understanding the fundamentals of teleophthalmology, our findings seem to align with past findings while offering the value of context in the subsequent follow-up phase. Furthermore, we had adjusted the ICO/ADA guidelines in hopes of minimizing false negatives. While current recommendations do not necessarily require referral to a specialist for cases of suspected mild non-proliferative DR [25], this program recommended referrals for all cases of suspected DR on screening. Not only are these recommendations comparable to those followed in previous teleophthalmology studies in other settings [27], but these recommendations granted some advantages relevant to patient care. For instance, the limited view and gradeability of our images may warrant concern for potentially missing false negative moderate-severe cases of DR that require more immediate referrals as per the ICO/ADA guidelines [25]. Given our awareness of the technological limitations and our later appreciation of the gradeability and limited view concerns, it was important that even suspected mild cases of DR be investigated further to limit missed cases of moderate-severe DR. As our prevalence data revealed, a larger proportion of patients who followed up with ophthalmologists had confirmed cases of moderate, severe, and proliferative DR. We also found that out of the population with exclusively mild DR suspected on at least one screening image, 48% completed follow-up and 5% of these patients were noted to have moderate, severe, or proliferative DR and/or the presence of DME—providing perhaps some support for these recommendations in the given context. Still, 36% of these patients with suspected mild DR had at least one eye with mild DR exclusively, and 59% had bilateral absence of DR/DME. However, with only 56% of suspects for moderate or severe non-proliferative DR, PDR, and/or DME following up (52.6% of DR suspects for all severities), it is apparent that the referral process and patient adherence are important areas in need of improvement for this program and should be key points to consider for other hospital systems hoping to adopt similar programs.

It is also paramount to consider that the technology involved in teleophthalmology is constantly evolving. Automation based on artificial intelligence has been proving its effectiveness in recent studies [11]. Likewise, upgrades to imaging technologies has also been promising. We utilized handheld, nonmydriatic cameras to take 45-degree images, but newer systems could grant specialists improved field of view and resolution. Ultrawide field technology, for instance, has shown notable success [38]. According to the findings of Silva et al., ultrawide field imaging technology has been shown to reduce the number of ungradable eyes by 81% [15, 32]. Improved field of view is also important for accurate disease interpretation. In addition to retinopathy, these improvements have implications for the use of telemedicine in addressing other pathology as well. In our study, we noted an abundance of other ocular pathologies, including age-related macular degeneration, hypertensive retinopathy, glaucomatous optic nerves, and choroidal nevi. Improvements in imaging would certainly benefit the identification of other pathologies as well.

Our teleophthalmology program hopes to make upgrades to the imaging technologies we utilize in order to improve the outcomes we observed in our current study and hopefully draw comparisons in the future. However, several important obstacles remain. Regardless of the improvements we achieve in our screening methods, the outcomes could fall short if patient compliance to follow-up does not improve. We suspected that the unique rural setting of West Virginia could play a role in this, and we found a statistically significant difference among patients who resided within 25 miles of the WVU Eye Institute and those who resided beyond 25 miles (60% versus 43%, respectively, *p* < 0.01). A potential limitation of this study entails possible follow-up with external providers throughout the state. Some patients’ providers had uploaded documentation regarding outside care. For others, we managed to find documentation mentioning ophthalmologists outside WVU Medicine. With permission and when feasible, we acquired documentation regarding follow-up visits from these outside offices. Unfortunately, there were likely still many patient follow-up appointments that were missed. Nevertheless, access to specialized care is a challenge for patients, and patient compliance to follow-up appointments may not be an uncommon issue amongst telemedicine programs. For instance, Peavey et al. found poorer follow-up among socioeconomically disadvantaged patients with milder DR severities in a predominantly rural population [39]. As mentioned previously, Bresnick et al. noted similar drops in compliance with plans to implement systems to hasten the delivery of results, improve engagement with patients when explaining their results and the implications they have for their vision, and reducing the window between result delivery and referral placement [29].

While we found numerous studies conducted in the context of urban settings [10, 16, 18, 23, 27, 28] and found common ground with socioeconomic obstacles [8, 9], we find our investigations of this statewide program that involves a rural setting to be unique and possibly useful to other programs. Teleophthalmology programs aiming to connect diabetic patients with specialist care through more accessible, feasible, efficient, and cost-effective screening approaches have the opportunity to improve outcomes for an ever-growing population of patients that is at risk of sight-threatening pathology. However, there are numerous obstacles to consider—one being the inherent geographic concern that is especially relevant to rural areas. New or current programs operating under similar circumstances might find a basis for comparison in our findings to set expectations and begin the process of addressing the next steps that follow the screening—ascertaining that correctly identified patients adhere to follow-up. Preferably, this can be accomplished within the hospital system or with external providers who can ensure the pipeline from screening to appropriate care is not broken. Working on methods to improve access to specialist care in WV and optimize/standardize the process of scheduling appointments for those identified by our screening to need dilated eye exams (standardizing PCP protocols/education and tracking these referrals and subsequent adherence) will be an important challenge to address as we seek to maximize the benefit of this teleophthalmology system and the quality of care it promotes.

## Conclusions

The success of teleophthalmology is contingent on a variety of factors. Many of these factors, such as age and distance from specialist care, were explored in this real-world application of teleophthalmology. These factors may be especially impactful in a rural setting, but they may also be applicable to teleophthalmology programs in other settings. While the implementation of telehealth technologies has facilitated the expansion of effective screening, follow-up confirmation of suspected diagnoses and appropriate initiation of treatment may remain hindered. This was especially suggested by the negative relationship we noted between distance from specialists and follow-up compliance among our patient population. The screening methods and statewide implementation of the program thus far among participating PCP sites has enabled extensive screening and identification of pathology. Improvements in equipment may also be promising for enhancing the accuracy of these screening approaches and possibly improving our image gradeability concern for the aging population. However, in order to improve outcomes in DR/DME patients and diabetic patients at risk of developing these sight-threatening pathologies, providers and program developers should be cognizant of the limitations brought about by geography and lack of convenient access to specialist care.

## Data Availability

The datasets used and analyzed in the current study are available from the corresponding author upon reasonable request.
